# An interdisciplinary integrated specialized one-stop outpatient clinic for idiopathic intracranial hypertension – an assessment of sick leave, presenteeism, and health care utilization

**DOI:** 10.1186/s10194-024-01780-9

**Published:** 2024-05-07

**Authors:** Gabriel Bsteh, Stefan Macher, Nik Krajnc, Wolfgang Marik, Martin Michl, Nina Müller, Sina Zaic, Jürgen Harreiter, Klaus Novak, Christian Wöber, Berthold Pemp

**Affiliations:** 1 Department of Neurology, Medical University of Vienna, Waehringer Guertel 18-20, 1090 Vienna, Austria; 2 Comprehensive Center for Clinical Neurosciences & Mental Health, Medical University of Vienna, Vienna, Austria; 3 Department of Neuroradiology, Medical University of Vienna, Vienna, Austria; 4 Department of Ophthalmology, Medical University of Vienna, Vienna, Austria; 5 Division of Endocrinology, Department of Internal Medicine, Medical University of Vienna, Vienna, Austria; 6 Department of Neurosurgery, Medical University of Vienna, Vienna, Austria

**Keywords:** Idiopathic intracranial hypertension, Neurology, Neuroophthalmology, Presenteeism, Economic, Outcome, Management, One-stop outpatient clinic

## Abstract

**Background:**

Management of idiopathic intracranial hypertension (IIH) is complex requiring contributions from multiple specialized disciplines. In practice, this creates considerable organizational and communicational challenges. To meet those challenges, we established an interdisciplinary integrated outpatient clinic for IIH with a central coordination and a one-stop- concept. Here, we aimed to evaluate effects of this concept on sick leave, presenteeism, and health care utilization.

**Methods:**

In a retrospective cohort study, we compared the one-stop era with integrated care (IC, 1-JUL-2021 to 31-DEC-2022) to a reference group receiving standard care (SC, 1-JUL-2018 to 31-DEC-2019) regarding economic outcome parameters assessed over 6 months. Multivariate binary logistic regression models were used to adjust for confounders.

**Results:**

Baseline characteristics of the IC group (*n* = 85) and SC group (*n* = 81) were comparable (female: 90.6% vs. 90.1%; mean age: 33.6 vs. 32.8 years, educational level: ≥9 years of education 60.0% vs. 59.3%; located in Vienna 75.3% vs. 76.5%). Compared to SC, the IC group showed significantly fewer days with sick leave or presenteeism (-5 days/month), fewer unscheduled contacts for IIH-specific problems (-2.3/month), and fewer physician or hospital contacts in general (-4.1 contacts/month). Subgroup analyses of patients with migration background and language barrier consistently indicated stronger effects of the IC concept in these groups.

**Conclusions:**

Interdisciplinary integrated management significantly improves the burden of IIH in terms of sick leave, presenteeism and healthcare consultations – particularly in socioeconomically underprivileged patient groups.

**Supplementary Information:**

The online version contains supplementary material available at 10.1186/s10194-024-01780-9.

## Introduction

Idiopathic intracranial hypertension (IIH; formerly also referred to as pseudotumor cerebri or benign intracranial hypertension) is a syndrome of increased intracranial pressure of unknown etiology [1]. Considered rare in the general population, IIH typically occurs in obese women of childbearing age with incidence increasing markedly due to the obesity pandemic [2, 3]. Main health associated risks of IIH include visual field loss and blindness if not treated in time, as well as disabling and often chronic headaches [4, 5]. The socioeconomic burden of IIH is also significant with estimated direct medical costs exceeding 444 million US dollars per year in the US alone (> 17.000 US dollars/patient) and massive secondary and tertiary costs assumed, mainly due to temporary or permanent disability [6].

Treatment of IIH should include a combination of weight loss, pharmacological treatment, and, in severe or refractory cases, invasive neurosurgical intervention [4, 7, 8]. Due to the increasing complexity of managing patients with IIH, international consensus guidelines recommend that IIH care should be provided in specialized centers with access to the necessary resources and therefore recommend interdisciplinary management of IIH [9, 10]. Despite this broad consensus, there are very few descriptions in the literature as to how such inter- or multidisciplinary management should be structured and organized in practice.

We have recently established an interdisciplinary integrated special outpatient clinic for IIH at our center providing a one-stop approach to diagnosis and treatment aiming to improve care.

Although such one-stop approaches are often promoted as a means of improving care, especially for chronic diseases with complex management, objective data on their outcome is very scarce. To date, there are no data on the explicit effects of interdisciplinary integrated care in IIH on sick leave, presenteeism, and health care utilization.

## Methods

This study was designed as a retrospective cohort study by analyzing the Vienna IIH Database (VIIH) of Department of Neurology, Medical University of Vienna, which is described in detail elsewhere [11]. As of September 30, 2023, the VIIH database contained a cohort of 289 patients with definite IIH according to the modified Friedman criteria [12]. VIIH case reports contain demographic data, details of diagnostic and therapeutic procedures as well as of the course of IIH.

### Study periods

Study periods covered the time from 1-JUL-2021 to 31-DEC-2022 for integrated care (IC) and 1-JUL-2018 to 31-DEC-2019 for standard care (SC). We chose two identical periods to minimize seasonal effects and we excluded the period from 1-JAN-2020 to 30-JUN-2021 to minimize direct and indirect influences of the SARS-CoV-2 pandemic and the measures to combat the pandemic.

### Intervention group: one-stop specialized interdisciplinary integrated care

**Fig. 1:**
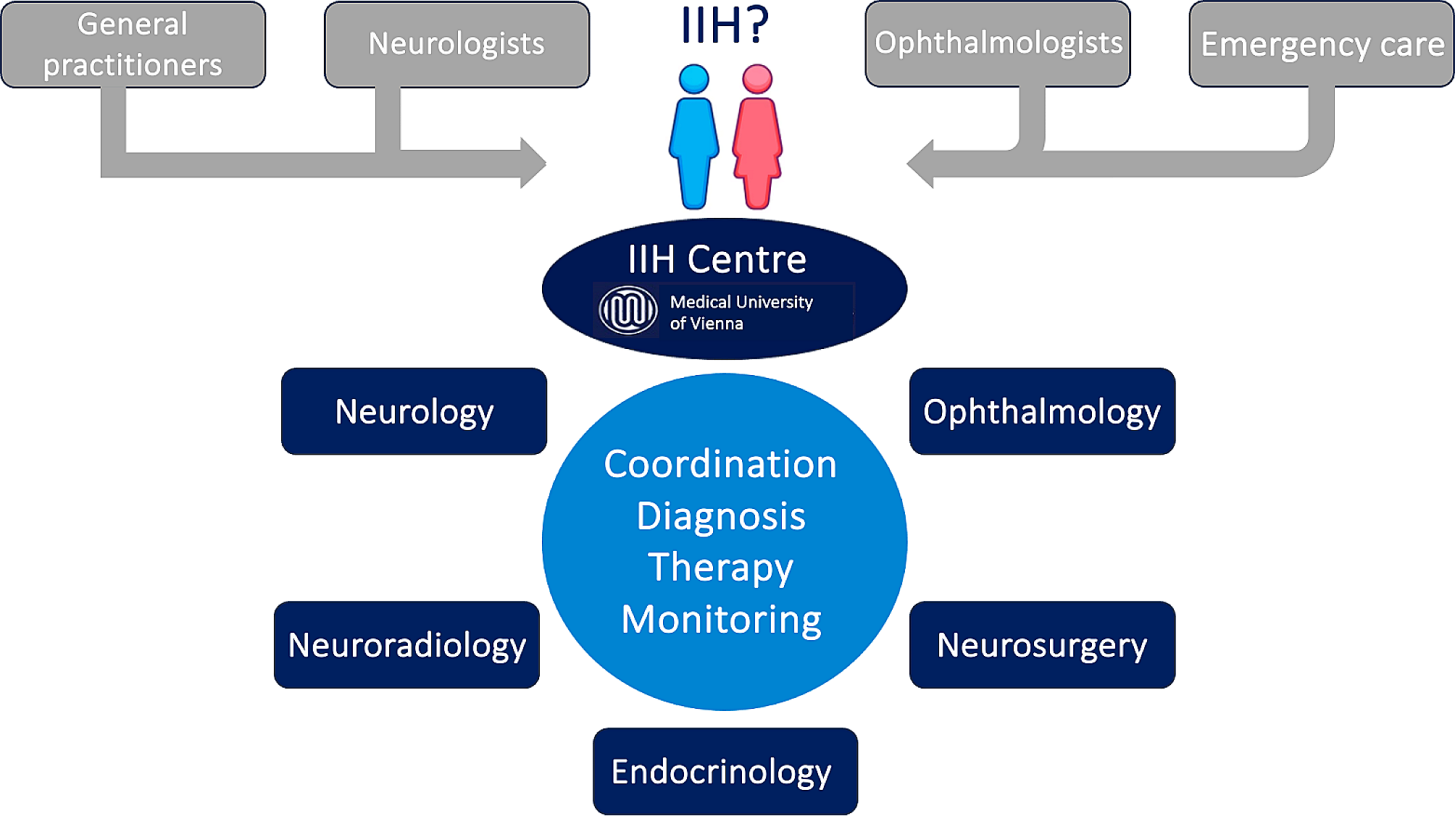
Structural process of the interdisciplinary integrative IIH outpatient clinic in Vienna

The interdisciplinary integrated IIH special outpatient clinic located at the Vienna General Hospital/Medical University of Vienna was established on April 1st, 2021. Outpatient care is provided in the outpatient clinics of Departments of Neurology, Neuroophthalmology and Endocrinology, and inpatient care at Department of Neurology and, if necessary, in Department of Neurosurgery (Fig. 1). Appointments for examinations and treatment are coordinated centrally (“one-stop approach”) by Department of Neurology and communicated to patients in a clear and structured manner in writing. Referrals from specialists in ophthalmology or neurology with a (suspected) diagnosis of IIH are received centrally and reviewed within 2 working days by a specialist from the IIH special outpatient clinic and an appointment for the first examination is made according to urgency. Without referral from an ophthalmologist or neurologist, patients can present themselves independently or on referral from a general practitioner at the general neurology outpatient clinic, from where they can be referred to the IIH special outpatient clinic. Visits are scheduled to last 60 min doctor-patient contact for the first presentation and 30 min for check-ups. The results of diagnostic processes and the choice of treatment options for patients of the IIH special outpatient clinic are discussed in a monthly interdisciplinary IIH board meeting chaired by neurology (comprising neuroophthalmology, neuroradiology, neurosurgery and endocrinology) and a joint recommendation is made. Necessary prescriptions for drug therapies are requested and issued by the IIH special outpatient clinic and given or sent directly to the patient. For patients with language barriers, a professional interpreter (either in person or via a video interpreting service) is used for all visits.

### Reference group – standard care

SC was assessed in the period before establishment of the IIH special outpatient clinic and required the patients to make appointments for clinical assessments, imaging and other instrumental examinations on their own without centralized coordination or comprehensive use of interpreters.

### Inclusion and exclusion criteria

We included all patients from the VIIH database with definite IIH according to the modified Friedman criteria and available follow-up of ≥ 6 months. To avoid censored data, patients for whom the period from initial visit to 6-month follow-up was either before the start or after the end of the defined time periods (01-APR-2018 to 30-SEP-2019 or 01-APR-2021 to 30-SEP 2022) were excluded.

### Economic outcome parameters

The primary economic endpoint was defined as the number of days of sick leave or days with impaired performance documented in the VIIH database on the basis of the medical history survey, whereby the monthly average during the first 6 months after diagnosis was used. In the case of permanent incapacity to work, the number of days of sick leave was assumed to be 30. For assessing healthcare utilization, we recorded the number of unscheduled physician contacts or hospital visits due to IIH-specific issues/problems (e.g. emergency room visits due to IIH-specific complaints, physician contacts for IIH-specific prescriptions) and the number of all physician contacts or hospital visits during the observation period.

### Covariates

Visual impairment was defined as a visual acuity deviation of ≥ 0.1 logarithm of the minimum angle of resolution (logMAR; determined by Sloan tables at distance after subjective refraction) and/or <-2.0 mean deviation in decibels (dB) in static threshold perimetry determined by the 30 − 2 Swedish Interactive Threshold Algorithm (SITA) [13]. Headache improvement was defined as a ≥ 50% reduction in headache severity (on the numerical analogue scale [NAS]) and/or headache frequency (determined by headache days per month) compared to baseline.

### Data curation and data analysis

The data relevant to this study were extracted from the VIIH database. The data contained in the VIIH database had already been regularly examined for outliers by two independent auditors (GB and PP). In addition, a random sample of 10% of the recorded patients was analyzed to confirm the quality of the original data collection. In order to further mitigate possible biases in the analysis of retrospective clinical data, a thorough quality control of the extracted data was carried out again for this study, in which the data was examined for outliers and a random sample of 5% of the recorded patients was re-evaluated entirely.

Statistical analyses were performed using R-Statistical Software (version 4.0.0). Univariate group comparisons were carried out as required using the chi-square test, the Fisher exact test, the Mann-Whitney U test or the independent t-test (with Welch correction for unequal standard deviations between the groups). Univariate correlation analyses were calculated using Pearson or Spearman-rho tests, depending on the presence of a normal distribution.

To investigate economic outcome, endpoints were initially compared univariately IC and SC. Subsequently, multivariate analyses using linear regression models with economic endpoints as the dependent variable and group affiliation as the independent variable (IC vs. SC) were performed. Corrected Akaike information criterion (AICc) was used to select the best-fitting model from a predefined set of known relevant covariates (age, gender, educational level [≤ 9 years of schooling vs. high school diploma/ university degree] and place of residence [Vienna vs. outside Vienna]) as well as all other variables associated with the endpoints with a p-value < 0.2 in univariate analyses [14]. Predefined subgroup analyses were conducted for patients with a language barrier (defined as German language proficiency ≤ B1) and patients with a first-generation migration background in order to explicitly examine the effects of integrated care on these potentially underserved patient groups. The robustness of all regression models to unidentified confounding factors (bias) was quantified using the Rosenbaum sensitivity test according to Hodges-Lehmann Gamma [15]. Missing values were treated by multiple (20-fold) imputation using the MNAR (Missing not at Random) approach with pooling of estimates according to Rubin’s rules [16]. Significance level was set at a two-sided p-value < 0.05.

### Standard protocol approvals, registrations, and patient consents

The study was approved by the ethics committee of the Medical University of Vienna (ethics approval number: 2216/2022). As this is a retrospective study, the ethics committee did not require a written declaration of consent from the study participants.

### Data availability

Data supporting the findings of this study are available from the corresponding author upon reasonable request by a qualified researcher and upon approval by the ethics committee and the data-clearing committee of the Medical University Vienna.

## Results

We included 85 patients in the IC group and 81 in the SC group. Characteristics of both groups are shown in Table 1. There were no significant differences between the groups at baseline, neither in terms of clinical nor demographic aspects.

**Table 1:** Baseline characteristics in integrated and standard care

	Integrated care (*n* = 85)	Standard care (*n* = 81)	*p*-value
Female^1^	77 (90.6)	73 (90.1)	0.999^4^
Age at diagnosis^2^	33.6 (9.8)	32.8 (10.3)	0.250^5^
Body Mass Index (BMI)^3^	31.8 (18.2–60.5)	33.0 (17.3–65.6)	0.523^6^
CSF opening pressure (cm H2O)^3^	33 (26–59)	31 (26–63)	0.422^4^
Papilledema grade (Frisen-scale)^3^	3 (0–5)	3 (0–5)	0.872^6^
Visual impairment at baseline^1^	61 (71.8)	56 (69.1)	0.736^4^
History of primary headache before diagnosis^1^	25 (29.4)	20 (24.7)	0.601^4^
History of migraine before diagnosis^1^	15 (17.7)	13 (16.1)	0.838^4^
Monthly headache days at baseline^3^	18 (0–30)	17 (0–30)	0.644^6^
Chronic headache^3^	47 (55.3)	46 (56.8)	0.877^4^
Headache severity (NRS)^3^	5.5 (0–10)	6.0 (0–10)	0.572^6^
Education level^1^			0.993^4^
≤ 9 years	34 (40.0)	33 (40.7)	
Highschool degree	29 (34.1)	27 (33.3)	
University degree	22 (25.9)	21 (25.9)	
Place of residence^1^			0.851^4^
Vienna	64 (75.3)	62 (76.5)	
Outside Vienna	21 (24.7)	19 (23.5)	
First generation migration background^1^	49 (57.7)	48 (59.3)	0.833^4^
Language barrier (level ≤ B1)	27 (31.8)	28 (34.6)	0.701^4^

NRS: numerical rating scale. ^1^absolute number (percentage). ^2^mean (standard deviation). ^3^median (range). ^4^calculated with chi-square test. ^5^calculated with t-test for independent groups. ^6^calculated with Mann-Whitney U-test.

**Fig. 2:**
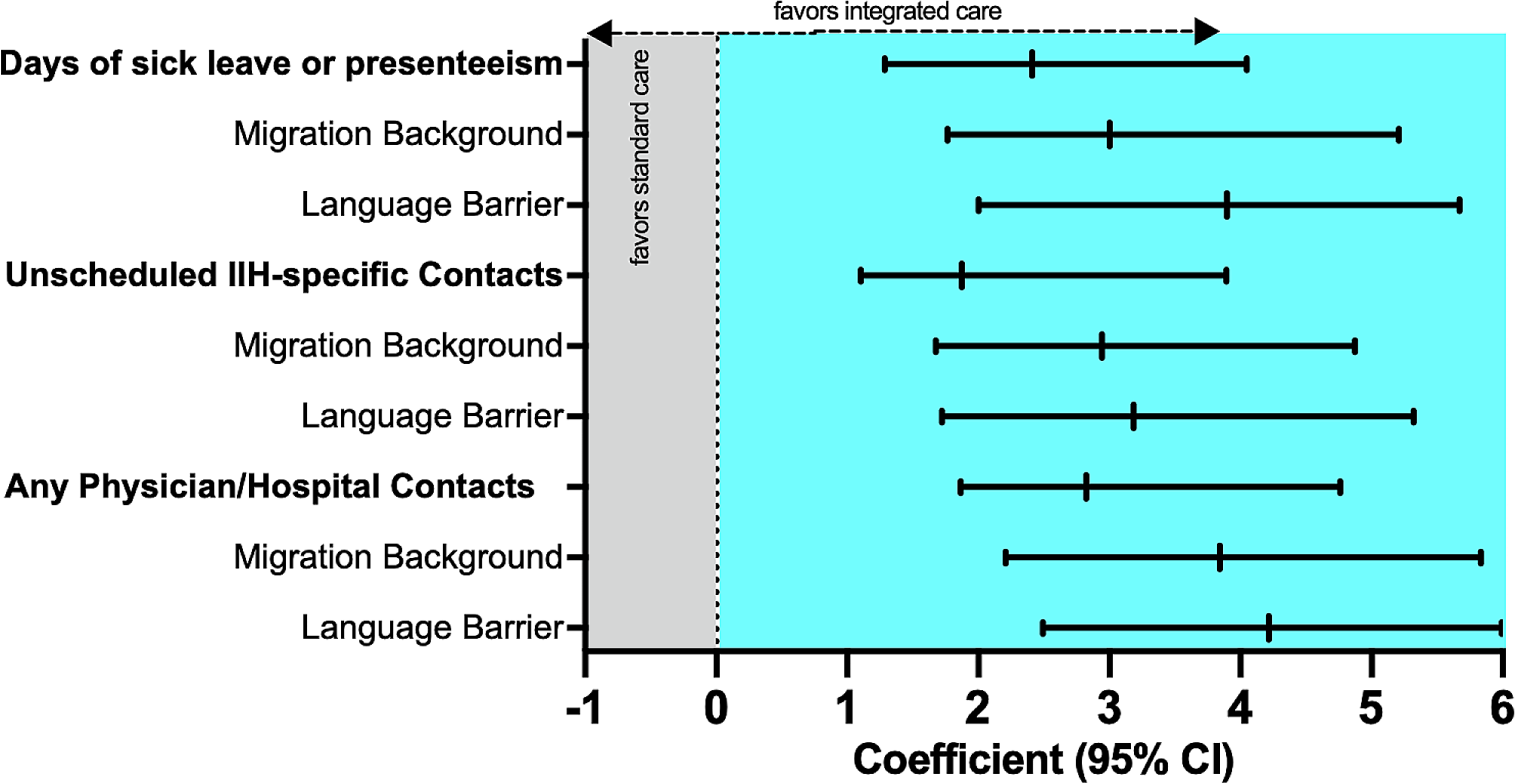
Impact of integrated care on sick leave, presenteeism, and health care utilization in the overall cohort and in subgroups with migration background and language barrier. ^a^calculated using linear regression models with economic outcome endpoints as the dependent variable and group affiliation as the independent variable (integrated specialized outpatient clinic vs. standard care). Positive values indicate a positive association with economic outcome

The average number of monthly days of sick leave or restricted performance, i.e. presenteeism, was significantly lower in the IC group with 6.9 days/month (SD 11.2) than in the standard treatment group with 11.9 days/month (10.1, *p* = 0.003). This was also observed in patients with migration background (7.6 vs. 15.2 days/month, *p* < 0.001) and with language barrier (7.4 vs. 19.0 days/month, *p* < 0.001, see Supplemental Table 1). In univariate analyses, the number of monthly days of sick leave or presenteeism was correlated with female sex (0.234, *p* < 0.001, lower educational level (-0.209, *p* = 0.021), visual impairment (0.443, *p* < 0.001) and lack of headache improvement (-0.672, *p* < 0.001). In the multivariate model, IC was significantly associated with a less days of sick leave/presenteeism with reference to SC (β=-2.412, *p* < 0.001, Table 2). The strength of association between IC and fewer days of sick leave or presenteeism was even higher in the subgroups with migration background (β=-3.003, *p* < 0.001) and with language barrier (β=-3.896, *p* < 0.001, Fig. 2). Female sex (β = 0.175, *p* = 0.036), visual impairment (β = 2.073, *p* < 0.001) and not achieving headache improvement (β = 4.135, *p* < 0.001) independently portended more days with sick leave or presenteeism (Table 2).

**Table 2:** Impact of integrated care compared to standard care on sick leave, presenteeism, and health care utilization in patients with IIH six months after diagnosis adjusted for relevant covariables

	Days of sick leave or presenteeism		
	β^a^	95% CI	*p*-value
Integrated care (vs. reference of standard care)	-2.412	-4.047 – -1.285	< 0.001
Female	0.175	0.010–0.321	0.036
Higher educational level^1^	-0.124	-0.426–0.087	0.099
Visual impairment at baseline	2.073	1.021–3.512	< 0.001
Headache improvement	-4.135	-6.831 – -2.296	< 0.001
	**Unscheduled IIH-specific contacts**		
Integrated care (vs. reference of standard care)	-1.872	-3.892 – -1.103	< 0.001
Female	0.203	0.086–0.345	0.012
Higher educational level^1^	-0.168	-0.476 – -0.002	0.049
Headache improvement	-4.231	-7.104 – -2.538	< 0.001
	**All physician contacts/hospital visits**		
Integrated care (vs. reference of standard care)	-2.824	-4.759 – -1.865	< 0.001
Higher educational level^1^	-0.238	-0.577 – -0.096	0.009
Headache improvement	-3.565	-5.819 – -2.004	< 0.001

^a^calculated using linear regression models with WPI scores as the dependent variable and group affiliation as the independent variable (integrated one-stop care vs. standard care). Positive values indicate a positive association of the respective variable with patient satisfaction

Corrected Akaike information criterion (AICc) was used to select the best-fitting model from known relevant covariates and other variables that were associated with the respective outcome measure with a p-value < 0.2 in univariate analyses

^1^high school diploma/ university degree referenced against ≤ 9 years of schooling

^2^resident in Vienna referenced against residence outside of Vienna

In comparison to standard care, the IC group displayed a significantly lower number of unscheduled IIH-specific contacts (1.8 [3.2] vs. 4.1 [4.5] per month, *p* < 0.001) and all physician contacts/hospital visits (2.7 [4.4] vs. 6.8 [5.9] per month, *p* < 0.001). Unscheduled IIH-specific contacts and all physician contacts/hospital visits were also significantly lower in the subgroups of patients with migration background and language barrier (Supplemental Table 1). Multivariate analyses showed strong associations of IC with fewer unscheduled IIH-specific contacts (β=-1.872, *p* < 0.001) and fewer all physician contacts/hospital visits compared to standard care (β=-2.824, *p* < 0.001) after adjusting for covariates (see Table 2). Analyzing the subgroups with migration background and language barrier revealed that the reduction of unscheduled IIH-specific contacts (β=-2.943 and β=-3.184, *p* < 0.001, respectively) and all physician contacts/hospital visits (β=-3.844 and β=-4.215, *p* < 0.001, respectively) in the IC group not only remained significant but showed significantly stronger effect sizes (Fig. 2). A higher educational level and achieving headache improvement both significantly conveyed fewer unscheduled IIH-specific and all physician contacts (Table 2).

## Discussion

Aiming to describe the effects of one-stop specialized interdisciplinary integrated care for IIH, on sick leave/presenteeism, and health care utilization as a means of economic outcome, we found that the one-stop concept showed significantly fewer days on sick leave/presenteeism (-5 days/month), fewer unscheduled contacts for IIH-specific problems (-2.3/month), and fewer physician or hospital contacts in general (-4.1/month). Importantly, subgroup analyses of patients with migration background and language barrier consistently indicated stronger effects of integrated care in these socioeconomically underprivileged groups.

In the literature, there is only one comprehensive description of an inter- or multidisciplinary organizational structure for IIH patients, which is established at the Danish Headache Center in Copenhagen [8]. There are several descriptions of multidisciplinary treatment protocols for IIH, which unanimously advocate the involvement of various specialist disciplines rather than care provided by a single discipline [10, 17–22]. Some protocols are limited to neurology, (neuro)ophthalmology and neurosurgery to identify patients whose visual function is acutely at risk [10, 20, 21]. Others recommend the additional involvement of secondary disciplines or health care professions to address other relevant aspects of IIH, e.g. nutritional counseling and physiotherapy to support weight loss or concomitant psychological and/or psychiatric care to treat patients’ comorbidities such as depression or eating disorders [8, 9, 18, 22, 23]. A one-stop structure for IIH, such as the Vienna Interdisciplinary Integrated Specialized Outpatient Clinic for IIH, has not yet been described in the field of IIH. Although inter-/multidisciplinary management of IIH is generally recommended, there are no data on the explicit effects of inter-/multidisciplinary structures of IIH care on patient satisfaction and economic aspects.

Studies of economic aspects place the success of medical services or treatments in relation to the financial and human resources used to in some way justify them in the context of the general scarcity of resources in the healthcare system [24, 25]. Parameters frequently used for this purpose are periods of absence from work or presenteeism [26].

The present study showed that, compared to standard care, patients receiving care in the one-stop concept spent significantly fewer days on sick leave or with reduced productivity (on average 5 days less per month), primarily by reducing the number of unscheduled contacts for IIH-specific problems but also physician or hospital contacts in general. While our study does not allow to draw definite conclusions on the specifically underlying reasons, we hypothesize that the main factors are (i) the central coordination of appointments within the one-stop approach effectively reducing the number of days patients had to take off from work for medical appointments, (ii) the comprehensive management covering most relevant aspects of patients with IIH reducing the need for “extracurricular” visits, and (iii) a faster implementation of treatment plans.

There are only a few studies in the literature that deal with economic aspects of care for IIH patients. None of these directly examined the effects of one-stop stores or integrative multi-/interdisciplinary care on economic aspects. However, a study at a Spanish tertiary hospital was able to show that establishing a multidisciplinary follow-up protocol significantly reduced the rate of necessary invasive therapies, from which a cost reduction can be derived at least indirectly, although no cost analysis was carried out [17]. A large study analyzing US insurance and registry data estimated direct and indirect healthcare costs caused by IIH to exceed 444 million US dollars per year in 2007 [6]. Hospital costs per IIH admission were about four times higher than with a population-based reference admission, which is even more relevant given the probability of hospitalization was 38% per year. Although these figures from the US healthcare system cannot be directly applied to Europe due to the completely different employment and insurance structure, it is clear to see that IIH represents an enormous financial burden for patients themselves as well as for the healthcare system. A follow-up study based on the same data sources showed that the probability of visiting an emergency room was not unexpectedly highest for newly diagnosed patients [27]. In our cohort, the probability of unscheduled physician/hospital contacts was slightly increased in women and patients with a lower level of education. However, the frequency of unscheduled contacts was much more strongly influenced by headache outcome, with patients with no improvement in headaches unsurprisingly requiring a significantly higher number of unscheduled contacts. However, these frequencies could be reduced by an average of 2.3 contacts per month through care in the one-stop setting, halving the frequency after adjustment for other factors. Since the frequency of all (i.e. regardless of the reason for the consultation) physician contacts was also significantly lower in the IC group, a reduction in costs can also be expected in addition to the obvious benefits for the quality of life of patients and the relief of emergency rooms and other structures that lack specialization for IIH. However, the costs associated with IIH management can vary widely depending on factors such as the severity of the condition, the individual patient’s needs, geographic location, healthcare system, insurance coverage, and specific treatment approaches [7, 28, 29]. In our practice, the one-stop outpatient clinic essentially assumed a hub function for IIH patients in the healthcare sector in the sense of a “quasi general practitioner”.

Beyond the overall paucity of data on the economic aspects of managing IIH patients, a look at other diseases that are comparable to IIH in certain aspects reveals a similar picture. A multidisciplinary one-stop outpatient clinic for migraine in San Diego, USA, was able to significantly reduce the frequency of contacts to primary care facilities or emergency rooms [30]. Studies on multidisciplinary one-stop in the UK and the US targeting patients with chronic back pain and multimorbidity, respectively, which similarly to IIH convey a high risk of repeated unplanned contact with healthcare facilities, were reported to significantly reduce such unscheduled contacts [31, 32].

The analysis of economic aspects in this study also showed that patients with a migration background and language barrier had significantly higher frequencies of sick days, unscheduled IIH-specific contacts and general contacts than the overall cohort in standard care. Here, we assume that these socio-economically underprivileged groups are disadvantaged in the standard care concept, which is probably due to a mixture of communication problems as a result of the language barrier and a fundamentally poorer understanding of the processes in the Austrian healthcare system among patients with a migrant background. This is consistent with US insurance data, where non-white IIH patients with low income had a higher risk of seeking emergency room care regardless of their clinical profile, again indicating a socioeconomic imbalance. Encouragingly, our study showed comparable frequencies of sick days, unscheduled IIH-specific contacts and general contacts in subgroups with migration background or language barrier when receiving IC.

### Limitations

The retrospective design of the study entails a number of limitations. The lack of randomization may induce several biases, e.g. a selection bias in the sense of an unbalanced selection of patients in a treatment group. However, this is mitigated by the VIIH database, which includes most IIH patients from our geographical area, and the very unselective inclusion criteria [11, 33]. Comparing patients from different time periods could theoretically lead to a systematic bias of the mean shift (Will-Rogers phenomenon), e.g. due to changes in the diagnostic and treatment processes or an immortality-of-time bias [33, 34]. This is particularly relevant because the SARS-CoV-2 pandemic and the measures to combat the pandemic lie between the investigated period of standard care and that of the intervention group. The comparison period for SC was chosen to minimize the direct and indirect influences of the SARS-CoV-2 pandemic and the measures to combat the pandemic. However, it is possible that patient perception and behavior regarding use of medical services may have changed as a result. Still, Rosenbaum sensitivity tests with Hodges-Lehmann Gamma indicated robustness to bias by unidentified confounders [15]. Finally, we need to acknowledge that due lack of data availability on patients’ income, quality of life and costs for the SC group, it was not possible to calculate direct and indirect health care costs.

In conclusion, the present study conducted in a representative and large (considering the rarity of IIH) sample of pwIIH shows that one-stop interdisciplinary integrated care independently improves economic outcome – particularly in socioeconomically underprivileged patient groups with migration background and/or language barrier.

Providing structured central coordination to facilitate and improve access to interdisciplinary management provides means to further improve outcome. This is deemed especially relevant, as over 90% of patients with IIH currently do not have access to inter-/multidisciplinary care [35]. Our data can be leveraged in the interaction with stakeholders and decision-makers to ensure that IIH patients are provided with the best possible care in the most efficient way.

### Electronic supplementary material

Below is the link to the electronic supplementary material.

Supplementary Material 1

## Data Availability

Data supporting the findings of this study are available from the corresponding author upon reasonable request by a qualified researcher and upon approval by the ethics committee and the data-clearing committee of the Medical University Vienna.
