# Supplementary material for: An interdisciplinary integrated specialized one-stop outpatient clinic for idiopathic intracranial hypertension – an assessment of sick leave, presenteeism, and health care utilization
Source: J Headache Pain. 2024 May 7;25(1):73. doi: 10.1186/s10194-024-01780-9 (PMC11075215; doi:10.1186/s10194-024-01780-9)
Supplement: Supplementary file 1 — Supplementary Material 1 [file 10194_2024_1780_MOESM1_ESM.docx]

**Supplemental Table 1. Subgroup analyses for patients with migration background and language barrier of economic outcome with integrated care for IIH and standard care.**

|  | **Migration background** | | | **Language barrier** | | |
| --- | --- | --- | --- | --- | --- | --- |
|  | **Integrated care (n=49)** | **Standard care (n=48)** | **p-value^2^** | **Integrated care (n=27)** | **Standard care (n=28)** | **p-value^2^** |
| **Sick leave or presenteeism (days/month)^1^** | 7.6 (10.9) | 15.2 (11.2) | <0.001 | 7.4 (11.4) | 19.0 (14.2) | <0.001 |
| **Unscheduled IIH-specific doctor contacts/hospital visits (days/month)^1^** | 2.1 (3.5) | 5.6 (6.7) | 0.002 | 2.3 (3.9) | 6.7 (7.1) | 0.007 |
| **All doctor contacts/hospital visit (days/month)^1^** | 3.1 (4.4) | 7.1 (8.5) | 0.005 | 3.2 (4.8) | 7.1 (8.8) | 0.046 |

^1^mean values and standard deviation. ^2^calculated by independent t-test.
